# Multiple lifestyle behaviours and mortality, findings from a large population-based Norwegian cohort study - The HUNT Study

**DOI:** 10.1186/s12889-016-3993-x

**Published:** 2017-01-10

**Authors:** Steinar Krokstad, Ding Ding, Anne C. Grunseit, Erik R. Sund, Turid Lingaas Holmen, Vegar Rangul, Adrian Bauman

**Affiliations:** 1 HUNT Research Centre, Department of Public Health and General Practice, Norwegian University of Science and Technology, Forskningsveien 2, 7600 Levanger, Norway; 2 Levanger Hospital, Nord-Trøndelag Hospital Trust, Levanger, Norway; 3 Prevention Research Collaboration, Sydney School of Public Health, The University of Sydney, Camperdown, NSW Australia; 4 Centre for Chronic Disease Prevention, College of Public Health, Medical and Veterinary Sciences, James Cook University, Cairns, QLD Australia

**Keywords:** Lifestyle behaviour, Risk factors, All-cause mortality, Cardiovascular disease, Metabolic disease, Cohort study

## Abstract

**Background:**

Lifestyle risk behaviours are responsible for a large proportion of disease burden and premature mortality worldwide. Risk behaviours tend to cluster in populations. We developed a new lifestyle risk index by including emerging risk factors (sleep, sitting time, and social participation) and examine unique risk combinations and their associations with all-cause and cardio-metabolic mortality.

**Methods:**

Data are from a large population-based cohort study in a Norway, the Nord-Trøndelag Health Study (HUNT), with an average follow-up time of 14.1 years. Baseline data from 1995–97 were linked to the Norwegian Causes of Death Registry. The analytic sample comprised 36 911 adults aged 20–69 years. Cox regression models were first fitted for seven risk factors (poor diet, excessive alcohol consumption, current smoking, physical inactivity, excessive sitting, too much/too little sleep, and poor social participation) separately and then adjusted for socio-demographic covariates. Based on these results, a lifestyle risk index was developed. Finally, we explored common combinations of the risk factors in relation to all-cause and cardio-metabolic mortality outcomes.

**Results:**

All single risk factors, except for diet, were significantly associated with both mortality outcomes, and were therefore selected to form a lifestyle risk index. Risk of mortality increased as the index score increased. The hazard ratio for all-cause mortality increased from 1.37 (1.15-1.62) to 6.15 (3.56-10.63) as the number of index risk factors increased from one to six respectively. Among the most common risk factor combinations the association with mortality was particularly strong when smoking and/or social participation were included.

**Conclusions:**

This study adds to previous research on multiple risk behaviours by incorporating emerging risk factors. Findings regarding social participation and prolonged sitting suggest new components of healthy lifestyles and potential new directions for population health interventions.

## Background

Lifestyle risk behaviours, such as poor diet, smoking, and physical inactivity are responsible for a large proportion of disease burden and premature mortality worldwide [1, 2]. Risk behaviours tend to co-occur and cluster in populations [3], suggesting the need for an integrated multiple behaviour approach. Previous studies have applied an index (i.e., a weighted or unweight sum of risk factors) as an indicator for overall or cumulative lifestyle risk [4]. A meta-analysis by Loef et al. found that those with at least four health behaviours had a 66% reduced risk of all-cause mortality [5]. In addition to all-cause mortality [6], lifestyle indexes have shown clear relationships with cancer risk [7–12], cardiovascular incidence and mortality [12–16] and diabetes [17]. A recent study showed that combinations of risk factors had synergistic effects on all-cause mortality, where some risk combinations were more harmful than others [4].

To date, most studies include smoking, at-risk alcohol consumption, physical inactivity, and poor diet as key components of lifestyle indexes [5]. With evidence accumulating on new risk factors, lifestyle indexes may be revisited and updated to reflect the current evidence base [16]. Emergent risk factors include prolonged *sitting time* which may be a risk factor for dysmetabolic indicators [18, 19] and all-cause mortality [20]. Research has also shown that insufficient or excessive amounts of *sleep* are associated with increased risk of chronic disease and mortality [21–23]. In addition, measures of *social connectivity* and social capital appear to be related to population health, contributing beyond that attributable to traditional risk factors [24–27]. For example, a recent study identified the association between social relations, social support, and chronic systemic inflammation, as a precursor of major chronic diseases and biomarkers of ageing [28]. These three emergent behaviours, namely, prolonged sitting, sleep duration, and social participation could be examined in addition to the existing lifestyle risk factors in new approaches to profiling ‘lifestyle risk’.

## Methods

The aim of this study was to extend previous work around lifestyle indexes in a prospective population based cohort study in Norway. Specifically, to determine whether sleep, sitting time and social participation would contribute to the concept of ‘lifestyle risk’, we examined their associations with all-cause and cardio-metabolic mortality as 1) single risk factors, 2) components of a new lifestyle risk index, and 3) in unique combinations with other lifestyle risk behaviours.

### Sampling and procedures

The Nord-Trøndelag Health Study (HUNT) is a large population-based cohort study conducted in the Nord-Trøndelag County located in central Norway (www.ntnu.edu/hunt) [29]. To date, three waves of data collection have been conducted: HUNT1 (1984–1986), HUNT2 (1995–1997), and HUNT3 (2006–2008). The current study is based on HUNT2 [30]. All inhabitants of the county aged 20 years and above were invited to participate and 70% joined the study by completing self-administered questionnaires on lifestyles and health (*n* = 78 976, participation rate 70%).

After excluding those aged 70 and above (*n* = 24 730) because they completed a different questionnaire which did not include all questions on lifestyle behaviours, and those with missing value on at least one lifestyle behaviour question (*n* = 17 516), our final analytical sample was restricted to 19 556 men and 17 355 women aged between 20 and 69 years at baseline.

### Measures

#### Lifestyle behaviours

##### Smoking

Participants were asked whether they smoked cigarettes/cigar/cigarillo/pipe on a daily basis, and those who were current smokers were defined as ‘at risk’.

##### Alcohol consumption

Alcohol risk was assessed using the Cut-Annoyed-Guilty-Eyeopener (CAGE) questionnaire (one point each for answering ‘yes’ to ever: ‘feeling the need to cut down on drinking’; ‘annoyed by people criticizing drinking’; ‘feeling guilty about drinking’; and ‘needed a drink first thing in the morning as an eye-opener’)[31]. The CAGE questionnaire has been extensively used and validated as a screening instrument for alcohol-related problems [32], and those who scored ≥2 were defined as ‘at risk’.

##### Diet

Dietary risk was assessed from one question indicating use of butter/hard margarine for cooking as compared with vegetable oil, oil blend, and soft margarine (as recommended by the health authorities). Participants using any of the former were classified as ‘at risk’. As only limited dietary questions were included in this survey, we used the current question as an indicator for dietary choice.

##### Physical activity

Physical activity was derived from two items measuring leisure-time ‘light’ activity (no sweating or being out of breath) and ‘hard’ activity (sweating/out of breath) during an average week in the last year. The ‘hard’ physical activity question has been found to be a valid measure of vigorous intensity physical activity [33, 34], and the ‘light’ physical activity question has moderate correlation with the International physical activity questionnaire (IPAQ) measure of moderate-intensity physical activity [33]. Being ‘at risk’ was defined as less than three hours/week of light and no hard activity or less than one hour per week of light and less than one hour per week of hard activity per week [35]. Missing values for one form of physical activity (but not both) (*n* = 16 595) were given zero for that physical activity type.

##### Sedentary behaviour

Sedentary behaviour was assessed using the single question ‘how many hours do you usually spend sitting down during a 24 h period?’ Participants were prompted to recall sitting at work, mealtimes, watching TV, sitting in a car, etc. We classified sitting for more than seven hours per day as ‘at risk’, based on a recent meta-analysis on sitting and all-cause mortality [36].

##### Sleep

Sleep time included both night time sleep and naps and we defined six hours/day or less (‘short duration’) and 10 h/day or more (‘long duration’) as ‘at risk’, based on recent meta-analytic evidence [37, 38].

### Social participation

Social participation was measured using a single item-question ‘How often do you usually participate in social activities such as a sewing club, athletic club, political association, religious or other groups?’ Those reporting ‘never, or only a few times a year’ were classified as ‘at-risk’ [27, 39].

### Mortality outcomes

The study data were linked to the Norwegian Causes of Death Registry by Statistics Norway, based on the unique Norwegian ‘personal identity number’, which is assigned to every Norwegian citizen at birth. Death information was obtained from 1 January 1994 to 31 December 2010, with endpoints as mortality from all causes and from cardio-metabolic diseases (CMD). Causes of death were coded based on the International Classification of Disease (ICD) and we identified deaths from CMD (diseases of the circulatory system, and endocrine, nutritional and metabolic diseases) using ICD-9 (codes 240–279, 390–459) for deaths occurring up to 1996 and ICD-10 (codes E10-E16, E65-E68, I00-I99) for deaths from 1996 onwards. Each participant contributed person years from the date of participation in the survey until the date of death or until the end of follow-up, whichever came first.

### Statistical analysis

The relationships between the seven lifestyle risk behaviours and mortality were analysed using Cox proportional hazards models for both all-cause and CMD mortality. Age was the time scale used with age at screening as the entry time and age at death/censoring as the exit time. Age rather than time-on-study was used as the time scale, as previous simulation studies have shown that the former method yields more accurate results because risk estimates are calculated on people of the same age [40]. Cox regression models were run firstly with each risk factor alone and then adjusted for sex, highest level of education attained and marital status as covariates. Competing risks models were used for CMD mortality to account for those dying of other causes [41]. Results are presented as adjusted hazard ratios (HR) for all-cause mortality and subhazard ratio (SHR) for CMD mortality.

Secondly, a lifestyle risk index was created by summing risk behaviours that were significant from the analysis described above. Poor diet was excluded from the index as none of the bivariate analyses demonstrated a significant association with all-cause or CMD mortality. All individual risk behaviours were coded 1 as ‘at risk’, and 0 as ‘not at risk’; therefore the overall index score ranged 0 to 6. Two Cox-regression models adjusted for sex, education and marital status were then run to examine the association between lifestyle risk index and all-cause and CMD mortality as described above.

Finally, we explored the implications for mortality of all 64 possible mutually exclusive combinations of the six components of the lifestyle risk index. We present and focus our interpretation on the most common combinations (occurring in >3% of the sample) due to the small cell sizes and wide confidence intervals in less common combinations. Based on Cox proportional hazards models, we calculated the hazard ratio and subhazard ratio for each combination compared with those without any risk factor [42]. All analyses were conducted using Stata 13.1 (College Station, TX, Stata Corporation) and a threshold of 0.05 used for statistical significance.

## Results

We linked 11 766 records of death, of which 5140 were caused by CMD, to 78 975 participant records from HUNT2 (excluding two participants for whom dates of death preceded their date of screening). The final sample for analysis included 36 911 participants who had complete data for all risk factors. They were aged between 20 and 69 at baseline, and 2220 had died (683 deaths caused by CMD) prior to 31 December 2010. The study sample had an average follow-up of 14.1 years for a total of 522 182 person years. At baseline, the average age of the participants was 43.6 years (SD = 12.9), 53% were women, 11.4% had an educational level of university qualifying exam/ junior college, and 24.7% had university/post-secondary education. Sixty-two percent of the sample was married or in a de-facto relationship.

The data shown in Table 1 suggests that the more prevalent risk factors were poor diet (63%), low social participation (41.4%), physical inactivity (40.1%) and prolonged sitting (38.7%), with fewer at risk from other factors (13.8% reported too little/too much sleep and 8.3% alcohol risk). All risk factors yielded highly significant (*p* < 0.001) differences across all of the demographic variables with the exception of poor diet by marital status (*p* = 0.527), probably due to the large sample size in the case where difference were small (i.e., less than 3%). However, a number of differences were larger and worthy of note. Overall, men had higher risk for alcohol-related problems, low social participation, and lower risk for physical inactivity than women. Those who were older had lower risk for smoking, alcohol, and excessive sitting but had higher risk for physical inactivity, and short/long sleep durations. Those with university education had lower risk for poor diet, smoking, physical inactivity, and low social participation, but were at higher risk for excessive sitting than those without a university education. Those who were married or in a de-facto relationship were at lower risk for alcohol-related problems, smoking, and poor social participation.

**Table 1:** Baseline demographic characteristics of adult respondents in the Nord-Trøndelag Health Study (the HUNT Study), Norway (1995–1997)

Characteristics^a^			Poor Diet	Alcohol risk	Currently Smoking	Physical inactivity	Excessive Sitting	Sleep risk	Poor social participation
		n	(%)^a^	(%)	(%)	(%)	(%)	(%)	(%)
Sex	Female	19 556	61.1	3.1	33.9	42.4	34.1	13.1	36.9
	Male	17 355	65.1	14.2	30.4	37.5	43.7	14.5	46.5
Age	20–35	10 697	64.6	9.5	29.6	31.4	39.1	15.4	40.7
	>35–50	14 217	62.9	9.1	36.5	41.4	41.1	11.8	36.8
	>50–65	9866	61.5	6.8	30.1	46.8	37.0	13.3	47.1
	>65–69	2131	62.7	4.7	26.3	44.3	27.6	21.2	49.5
Educational attainment	Primary school	9392	64.1	7.3	40.4	52.6	27.4	17.0	54.3
	High school	13 768	66.4	9.2	36.4	41.8	34.2	14.1	41.9
	Junior college	4203	64.0	8.6	27.3	31.0	46.7	13.4	34.6
	University/post-secondary	9126	56.3	8.0	19.8	28.5	53.3	9.9	30.0
	Missing	422	60.7	6.4	33.2	46.9	36.3	21.1	53.3
Marital status	Single/divorced/separated/windowed	14 113	63.3	11.4	35.9	36.4	40.2	16.2	47.3
	Married/De-facto	22 708	62.8	6.4	29.9	42.4	37.7	12.3	37.7
	Missing	90	60.0	6.7	30.0	41.1	47.8	14.4	50.0
Total	All respondents	36 911	63.0	8.3	32.2	40.1	38.7	13.8	41.4

^a^Poor diet: measured using a dietary indicator (i.e., using butter/hard margarine for cooking); Alcohol risk was measured using the CAGE questionnaire; current smoker; physical inactivity was defined as <3 h/week moderate PA and no vigorous PA OR <1 h/week moderate PA and <1 h/week vigorous PA/week; Excessive siting was defined as sitting for more than 7 h per day; sleep risk was defined as sleeping too little (≤6 h/day) or too much (≥10 h/day); poor social participation was defined as ‘never or only a few times per year’ of participation in social clubs, associations or groups

Table 2 presents the unadjusted and adjusted association between each individual risk factor and all-cause and CMD-mortality. All risk behaviours, except for poor diet, were significantly associated with both mortality outcomes in unadjusted models, and remained significant but slightly attenuated in adjusted models. Based on the adjusted models, smoking and alcohol risk had the strongest associations with all-cause mortality, and smoking and physical inactivity had the strongest associations with CMD mortality.

**Table 2:** Individual lifestyle risk behaviour associated with all-cause and cardio-metabolic disease (CMD) mortality in the Nord-Trøndelag Health Study (the HUNT Study), Norway (1995–1997 to 2010, *n* = 36 911)

		All-cause mortality				CMD mortality		
Risk Factor^a^		n	Total died	Unadjusted HR (95% CI)	Adjusted HR^b^ (95% CI)	Total died	Unadjusted SHR (95% CI)	Adjusted SHR^b^ (95% CI)
Diet	Not at risk	13 659	848	1	1	249	1	1
	At risk	23 252	1,372	0.99 (0.91-1.08)	0.96 (0.88-1.05)	434	1.07 (0.92-1.25)	1.04 (0.89-1.21)
Alcohol consumption	Not at risk	33 837	1,981	1	1	617	1	1
	At risk	3074	239	1.84 (1.61-2.10)	1.47 (1.28-1.68)	66	1.59 (1.24-2.06)	1.12 (0.86-1.45)
Smoking	Not at risk	25 016	1,204	1	1	387	1	1
	At risk	11 895	1,016	2.16 (1.99-2.35)	2.06 (1.89-2.24)	296	1.86 (1.60-2.16)	1.71 (1.47-1.99)
Physical activity	Not at risk	22 113	1,143	1	1	335	1	1
	At risk	14 798	1,077	1.19 (1.09-1.29)	1.23 (1.13-1.34)	348	1.27 (1.09-1.47)	1.35 (1.16-1.58)
Sitting time	Not at risk	22 646	1,389	1	1	408	1	1
	At risk	14 265	831	1.18 (1.08-1.28)	1.16 (1.06-1.27)	275	1.35 (1.16-1.58)	1.30 (1.11-1.52)
Sleep Duration	Not at risk	31 821	1,785	1	1	535	1	1
	At risk	5090	435	1.35 (1.22-1.50)	1.32 (1.19-1.47)	148	1.48 (1.23-1.78)	1.44 (1.19-1.73)
Social participation	Not at risk	21 631	1,015	1	1	294	1	1
	At risk	15 280	1,205	1.41 (1.30-1.53)	1.25 (1.15-1.37)	389	1.48 (1.28-1.73)	1.24 (1.06-1.45)

^a^Poor diet: measured using a dietary indicator (i.e., using butter/hard margarine for cooking); Alcohol risk was measured using the CAGE questionnaire; physical inactivity was defined as <3 h/week moderate PA and no vigorous PA OR <1 h/week moderate PA and <1 h/week vigorous PA/week; Excessive siting was defined as sitting for more than 7 h per day; sleep risk was defined as sleeping too little (≤6 h/day) or too much (≥10 h/day); poor social participation was defined as ‘never or only a few times per year’ of participation in social clubs, associations or groups. ^b^Adjusted Hazard Ratio (HR) and subhazardratio (SHR): Adjusted for sex, educational attainment, marital status

Due to the lack of association between dietary risk and mortality outcomes, we summed only the remaining six risk behaviours to create a lifestyle risk index. As Table 3 indicates, there was a dose–response relationship between the index score and mortality, and the association was particularly strong with CMD mortality. Those with more than three risk conditions showed a three to six-fold increase in the risk of all cause or CMD deaths, irrespective of which risk factors were present.

**Table 3:** Lifestyle risk index^a^ associated with all-cause and cardio-metabolic disease (CMD) mortality in the Nord-Trøndelag Health Study (the HUNT Study), Norway (1995–1997 to 2012, *n* = 37 785)

	All-cause mortality			CMD mortality	
	n	Deaths	Adjusted HR^b^ (95% CI)	Deaths	Adjusted SHR^b^ (95% CI)
No risk behaviour	5312	170	1	39	1
One	11 703	582	1.37 (1.15-1.62)	180	1.75 (1.24-2.48)
Two	11 364	673	1.55 (1.31-1.84)	210	1.94 (1.38-2.73)
Three	6557	566	2.26 (1.91-2.69)	184	2.80 (1.97-3.96)
Four	2312	260	3.16 (2.60-3.85)	84	3.66 (2.49-5.37)
Five	480	72	4.26 (3.22-5.62)	28	5.28 (3.18-8.77)
Six	57	14	6.15 (3.56-10.63)	2	2.23 (0.50-9.92)

^a^Lifestyle risk index was a sum of the following six risk behaviours: at-risk alcohol consumption, currently smoking, physical inactivity, excessive sitting, sleep too little/much, and poor social participation

^b^ *HR*, Hazard ratio and *SHR*, subhazardratio: Adjusted for sex, educational attainment and marital status

Table 4 shows the 12 most prevalent combinations of risk behaviours among the participants. Compared with those without any risk behaviour, which amounted to just over 14% of the sample, the most common risk profiles were ‘excessive sitting only’ (9.6%, no significant association with mortality), ‘physical inactivity only’ (7.5%, significantly associated with CMD mortality, adjusted HR = 1.68), ‘poor social participation’ (6.8%, significantly associated with all-cause mortality, adjusted HR = 1.42 and CMD-mortality, adjusted SHR 1.57), and ‘physical inactivity and poor social participation’ (5.4% no significant association with mortality). The largest effect size was for the combination was for smoking, physical inactivity and poor social participation, increasing the risk of all-cause mortality by a factor of 2.87 and CMD-mortality by 3.13 when compared with having no risk factors and adjusting for demographic characteristics. Smoking either alone or in combination was implicated in the four risk factor combinations yielding the highest hazards for all-cause mortality, and in three of the top four for CMD-mortality.

**Table 4:** The most common 12 combinations of individual risk behaviours associated with all-cause and cardio-metabolic disease (CMD) mortality in the Nord-Trøndelag Health Study (HUNT), Norway (1995–1997 to 2012, *n* = 37 785)

Combinations	Alcohol	Smoking	Physical inactivity	Sitting	Sleep	Social participation	All-cause mortality			CMD mortality	
							Prevalence	Deaths	Adjusted^a^ HR (95% CI)	Deaths	Adjusted^a^ SHR (95% CI)
1	_**−**_	_**−**_	_**−**_	_**−**_	_**−**_	_**−**_	14.1	170	1	39	1
2	_**−**_	_**−**_	_**−**_	_**+**_	_**−**_	_**−**_	9.6	114	1.15 (0.90-1.48)	33	1.46 (0.91-2.35)
3	_**−**_	_**−**_	_**+**_	_**−**_	_**−**_	_**−**_	7.5	131	1.18 (0.93-1.49)	41	1.68 (1.07-2.66)
4	_**−**_	_**−**_	_**−**_	_**−**_	_**−**_	_**+**_	6.8	177	1.42 (1.14-1.77)	58	1.57 (1.03-2.39)
5	_**−**_	_**−**_	_**+**_	_**−**_	_**−**_	_**+**_	5.4	123	1.14 (0.89-1.45)	32	1.00 (0.63-1.60)
6	_**−**_	_**−**_	_**−**_	_**+**_	_**−**_	_**+**_	4.5	68	1.14 (0.85-1.52)	25	1.61 (0.96-2.69)
7	_**−**_	_**+**_	_**−**_	_**−**_	_**−**_	_**−**_	4.5	111	2.30 (1.80-2.93)	29	2.40 (1.47-3.91)
8	_**−**_	_**−**_	_**+**_	_**+**_	_**−**_	_**−**_	4.4	85	1.47 (1.13-1.91)	30	2.22 (1.39-3.54)
9	_**−**_	_**+**_	_**+**_	_**−**_	_**−**_	_**+**_	3.8	161	2.87 (2.28-3.62)	47	3.15 (1.98-5.03)
10	_**−**_	_**+**_	_**−**_	_**−**_	_**−**_	_**+**_	3.6	124	2.19 (1.72-2.8)	38	2.31 (1.43-3.74)
11	_**−**_	_**+**_	_**+**_	_**−**_	_**−**_	_**−**_	3.3	77	2.17 (1.64-2.88)	23	2.77 (1.64-4.67)
12	_**−**_	_**−**_	_**+**_	_**+**_	_**−**_	_**+**_	3.2	83	1.56 (1.19-2.04)	35	2.69 (1.59-4.20)

^a^ *HR*, Hazard ratio and *SHR*, subhazardratio: Adjusted for sex, educational attainment and marital status

## Discussion

Lifestyle risk indexes can be used as parsimonious indicators of a person’s overall or cumulative risk for non-communicable chronic disease and mortality. We examined whether sleep, sitting time and social participation could be usefully added to existing life style indexes, and our results showed these risk factors captured additional risk of death in a population sample of Norwegian adults. All risk behaviours except for the diet indicator were significantly associated with both mortality outcomes even when adjusted for socio-demographic characteristics. Moreover, clusters of risk behaviours prevalent in the sample demonstrated the incremental effect on mortality as the total number increased in any combination, but particularly when including smoking and/or social participation.

The twelve most prevalent patterns of risk factor combinations shown in Table 4, described just over 70% of the sample. Prolonged sitting in isolation was not a significant risk factor, and physical inactivity by itself, was a risk factor for CMD only. When in combination together however, they represented 4.4% of the sample, and showed substantial increased risk for all cause and particularly CMD deaths. This finding is similar to that report in a recent study conducted in Australia [4]. Additionally, physical inactivity was implicated in half of the top twelve risk combinations in terms of prevalence signalling that a change in this specific risk factor would represent change in the mortality risk in over a quarter of the population.

Social participation and smoking, by themselves, or in combination with other risks, were strongly associated with all-cause and CMD deaths. The combinations of risk factors show that some are synergistic. Although public health interventions should target all risk factors, practically, an efficient approach might be an integrated chronic disease prevention strategy with a focus on sub-clusters, identified here, that are synergistic.

Both sleep duration and sleep quality have previously been related to mortality [38], work related disability [43], health care utilization and medication. Our data suggest that sleep should be a useful addition to include in a lifestyle risk index. Social relationships have also been shown to have powerful effects on health and survival [25, 27]. New evidence indicates that the bio-physiological mechanisms which underlie these links may involve altered immune functions [28] that can play a role in increased risk for many different chronic diseases. Our data are consistent with these findings. Significantly, both sleep and social participation are factors available for interventions at both the individual and the population level.

Risky alcohol consumption didn’t appear among the top twelve combinations in our material. Alcohol consumption was relatively low in Norway in the 1990s, but has increased considerably the last decades [44]. In Australia, risky alcohol consumption had higher impact on mortality in a comparable study [16].

### Strengths and limitations

The strength of this study is that the sample was from an unselected homogenous population in a defined geographical area in a northern European country. The validity of the main endpoint, i.e. death, is considered to be very accurate because of the high quality of national registries in Norway [45]. The response rate was relatively high, the sample size was satisfactory for most analyses, and follow-up time was sufficiently long.

Previous analyses from the HUNT Study have shown that those who declined to participate had somewhat lower socioeconomic status and slightly higher prevalence of chronic diseases and higher mortality than those who participated [46]. Further, we had to exclude a large number of participants because of missing values on at least one lifestyle risk factor. This could be particularly an issue for the current study because HUNT2 baseline measures were assessed through multiple questionnaires. A supplementary analysis comparing those with missing data on one or more risk factors by sex, age, education and marital status showed that older participants and those with lower education had higher rates of missing data than younger and more educated participants. Both of these variables have been included in the models, thereby providing model-based adjustment for the missing data [47]. Further, there is little reason to believe that this may have affected the association between lifestyle and mortality in this study significantly, as exposure-outcome associations based on internal comparisons are usually not dependent on the representativeness of the cohort [48]. The survey variables vary somewhat in quality and validity, especially for diet, where the question asked was limited, and therefore we probably have under-estimated the importance of diet. Substituting zero for missing data on the PA measures constituting the physical inactivity risk factor may have biased downwards estimates of those sufficiently active. However, supplementary analyses excluding these observations (*n* = 5135) changed the effect size and direction of the mortality results only minimally and statistical significance not at all. Although the analyses showed a clear relationship between the risk factors and mortality with an incremental increase in risk with an increasing number of any of these risk factors, the small sample reporting six risk factors limits the reliability for that number. Only a small number of people in the youngest age group (20–35 years) died of CMD (*n* = 19). Therefore, these and all results would benefit from further validation in other cohorts.

### Future directions

To refine this type of research, there is a need for better data on physical activity, more comprehensive data on diet and better characterization of other lifestyle behaviours. In the future, more objective measurements probably will become available for several types of behaviour, which can lead to more precise guides for public health efforts. A new wave of the HUNT Study (HUNT4) is being planned to be completed in 2017–2018. New objective measurements have high priority in this work.

## Conclusions

Our data showed the effects of several traditional and emerging risk factors alone and in concert with others. Sleep duration, sitting time and social participation should be added to existing lifestyle indexes to predict all-cause and CMD mortality [4, 16, 27, 49]. Clinicians should pay attention to new components in “healthy lifestyles”, but also be aware of the health services’ limitations in terms of reducing all-cause and CMD mortality in the population because of the reliance on high-risk strategies targeting individuals [49]. Healthcare workers could contribute to the understanding of the potential of population-based prevention strategies in the society, to reduce detrimental social living conditions contributing to unhealthy sleep patterns, increasing everyday sitting time and barriers to social participation.

## Abbreviations

CAGE: Cut-annoyed-guilty-eyeopener questionnaire
CI: Confidence interval
CMD: Cardio-metabolic diseases
HR: Hazard ratio
HUNT: The Nord-Trøndelag Health Study
ICD: International classification of disease
IPAQ: International physical activity questionnaire
SHR: Subhazard ratio
